# Ada2 and Ada3 Regulate Hyphal Growth, Asexual Development, and Pathogenicity in Beauveria bassiana by Maintaining Gcn5 Acetyltransferase Activity

**DOI:** 10.1128/spectrum.00281-23

**Published:** 2023-04-13

**Authors:** Shun-Juan Hu, Hao Zheng, Xin-Peng Li, Zhi-Xing Li, Chao Xu, Juan Li, Jia-Hua Liu, Wen-Xiao Hu, Xian-Yan Zhao, Juan-Juan Wang, Lei Qiu

**Affiliations:** a State Key Laboratory of Biobased Material and Green Papermaking, Qilu University of Technology, Shandong Academy of Sciences, Jinan, China; b School of Biological Science and Technology, University of Jinan, Jinan, China; The Ohio State University

**Keywords:** *Beauveria bassiana*, Ada2, Ada3, Gcn5 acetyltransferase activity, asexual development, pathogenicity

## Abstract

The histone acetyltransferase (HAT) Gcn5 ortholog is essential for a variety of fungi. Here, we characterize the roles of Ada2 and Ada3, which are functionally linked to Gcn5, in the insect-pathogenic fungus Beauveria bassiana. Loss of *Ada2* and *Ada3* led to severe hyphal growth defects on rich and minimal media and drastic decreases in blastospore yield and conidiation capacity, with abnormal conidia-producing structures. Δ*Ada2* and Δ*Ada3* exhibited a delay in conidial germination and increased sensitivity to multiple chemical stresses and heat shock. Nearly all their pathogenicity was lost, and their ability to secrete extracellular enzymes, Pr1 proteases and chitinases for cuticle degradation was reduced. A yeast two-hybrid assay demonstrated that Ada2 binds to Ada3 and directly interacts with Gcn5, confirming the existence of a yeast-like Ada3-Ada2-Gcn5 HAT complex in this fungus. Additionally, deletion of the *Ada* genes reduced the activity of Gcn5, especially in the Δ*Ada2* strain, which was consistent with the acetylation level of histone H3 determined by Western blotting. These results illustrate the dependence of Gcn5 enzyme activity on Ada2 and Ada3 in fungal hyphal growth, asexual development, multiple stress responses, and pathogenicity in B. bassiana.

**IMPORTANCE** The histone acetyltransferase Gcn5 ortholog contributes significantly to the growth and development of various fungi. In this study, we found that Ada2 and Ada3 have critical regulatory effects on Gcn5 enzyme activity and influence the acetylation of histone H3. Deletion of *Ada2* or *Ada3* decreased the fungal growth rate and asexual conidial yield and increased susceptibility to multiple stresses in Beauveria bassiana. Importantly, *Ada* genes are vital virulence factors, and their deletion caused the most virulence loss, mainly by inhibiting the activity of a series of hydrolytic enzymes and the dimorphic transition ability. These findings provide a new perspective on the function of the Gcn5 acetyltransferase complex in pathogens.

## INTRODUCTION

Beauveria bassiana is a typical entomopathogenic fungus with a wide range of hosts and has been applied extensively for the biological control of insect pests (1). In general, infection with B. bassiana in host insects involves several steps: conidial adhesion and penetration of the host cuticle, proliferation in the host body, inside-out growth, and penetration of the cuticle to produce conidia on the surface of the cadaver (2, 3). In this process, B. bassiana requires a variety of transcriptional regulatory mechanisms to control gene expression to adapt to changes in the environment (4). An important epigenetic regulatory mechanism, histone acetylation, has previously been shown to be highly related to transcriptional activation (5). Histone acetylation is performed by a class of histone acetyltransferases (HATs), which are responsible for transferring acetyl groups to lysine residues, making chromatin change into flexible structures for transcriptional activation (6).

Gcn5 (General control nonderepressible 5) is the first identified transcription-linked HAT with specificity for lysine residues on the N-terminus of histones H3 and H2B (7–9). In yeast, Gcn5 works as the catalytic core in several multisubunit protein complexes, including SAGA (Spt-Ada-Gcn5-acetyltransferase) and ADA (Ada2-Gcn5-Ada3) (10, 11). Genome-wide mapping analysis has revealed that the SAGA complex participates in the regulation of approximately 10% of the yeast genome, and the SAGA-dominated genes are mainly stress-induced by heat, oxidative stress, and starvation in Saccharomyces cerevisiae (12). Loss of *GCN5* leads to slow growth of yeast on minimal medium and increased sensitivity to cold and heat (13). In addition, Gcn5 also contributes to multiple processes in pathogenic fungi, including cell growth, development, and virulence. The absence of *GCN5* in Candida albicans is associated with defects in hyphal formation and virulence in a murine model, and *GCN5* regulates gene expression in multiple pathways required for fungal virulence and antifungal susceptibility (14, 15). In Aspergillus flavus, AflGcnE (Gcn5 homolog) plays an integral role in morphogenesis, aflatoxin biosynthesis, stress response, and pathogenicity (16).

The Gcn5-containing HAT complexes consist of a set of complex-specific proteins and several transcriptional adaptor proteins, such as Ada (alteration/deficiency in activation) 2 and Ada3 (17, 18). These proteins affect the Gcn5’s catalytic activity, substrate specificity, and the ability to regulate transcriptional activation through the acetylation of histones (19, 20). For example, recombinant Gcn5 protein alone can effectively acetylate free histones but not nucleosomal histone substrates *in vitro*. Gcn5 existing in the intact SAGA protein complex or with Ada2 and Ada3 has stronger HAT activity on both free and nucleosomal histones (21–23). Some *ada2* mutations in yeast cause an absence of the Gcn5 subunit in the SAGA complex, which impairs cell growth and transcription *in vivo* (24–26). C. albicans Ada2p is associated with fungal tolerance to oxidative stress and to treatments with tunicamycin and fluconazole (27). Meanwhile, the *ada2* deletion mutant showed hyphal growth defects and reduced virulence in mouse and Caenorhabditis elegans infection models (27, 28). Similarly, the *NGG1* gene (*Ada3* homolog) is essential for filamentous growth and pathogenicity in these two infection models and plays a critical role in the histone H3 acetylation in C. albicans (29). In Cryptococcus neoformans, the absence of *ADA2* leads to growth defects at high temperatures and influences capsule formation and fungal virulence (30). The Aspergillus nidulans homolog of Ada2, adaB, is required for growth, conidiation, and conidiophore formation (31). *Mrada3* is involved in sexual reproduction and the production of secondary metabolites in industrial fungi of the *Monascus* strain (32). Ada2 and Ada3 in Fusarium *fujikuroi* participate in the regulation of growth and conidiation and in secondary metabolite biosynthesis (33).

In B. bassiana, Gcn5 is able to acetylate the manifold targets on histone H3 and contributes to the activity of genes involved in asexual development, dimorphic transition, and virulence (34). However, the effects of Ada2 and Ada3 on Gcn5 and their roles remain unknown. In this study, we elucidated the role of Ada2 and Ada3 in B. bassiana via gene knockout and complementation. Ada2 and Ada3 modulate fungal hyphal growth, asexual development, stress resistance, and virulence by affecting Gcn5 enzymatic activity and histone H3 acetylation in B. bassiana.

## RESULTS

### Characterization and deletion of the *Ada2* and *Ada3* genes.

Using S. cerevisiae Ada2 and Ngg1 as queries for an online protein BLAST search, the two proteins were identified in the genome of B. bassiana and then named Ada2 (NCBI Accession: EJP69137.1) and Ada3 (NCBI Accession: EJP70893.1), respectively. In B. bassiana, the Ada2 and Ada3 proteins, respectively, consist of 514 and 630 amino acids and share 39.8% and 30.48% identity with their S. cerevisiae orthologs.

To uncover the functions of *Ada2* and *Ada3*, partial gene fragments were replaced with the *bar* gene through homologous recombination to generate Δ*Ada2* and Δ*Ada3* mutants. The complementation strains *Ada2*^C^ and *Ada3*^C^ were obtained by ectopic integration of the entire *Ada2* or *Ada3* gene into the corresponding deletion strain. PCR and quantitative real-time PCR (qRT-PCR) were run to verify the *Ada* deletion and complementation mutants (Fig. S1).

### Ada2 and Ada3 control fungal hyphal growth and stress tolerance.

**FIG 1:**
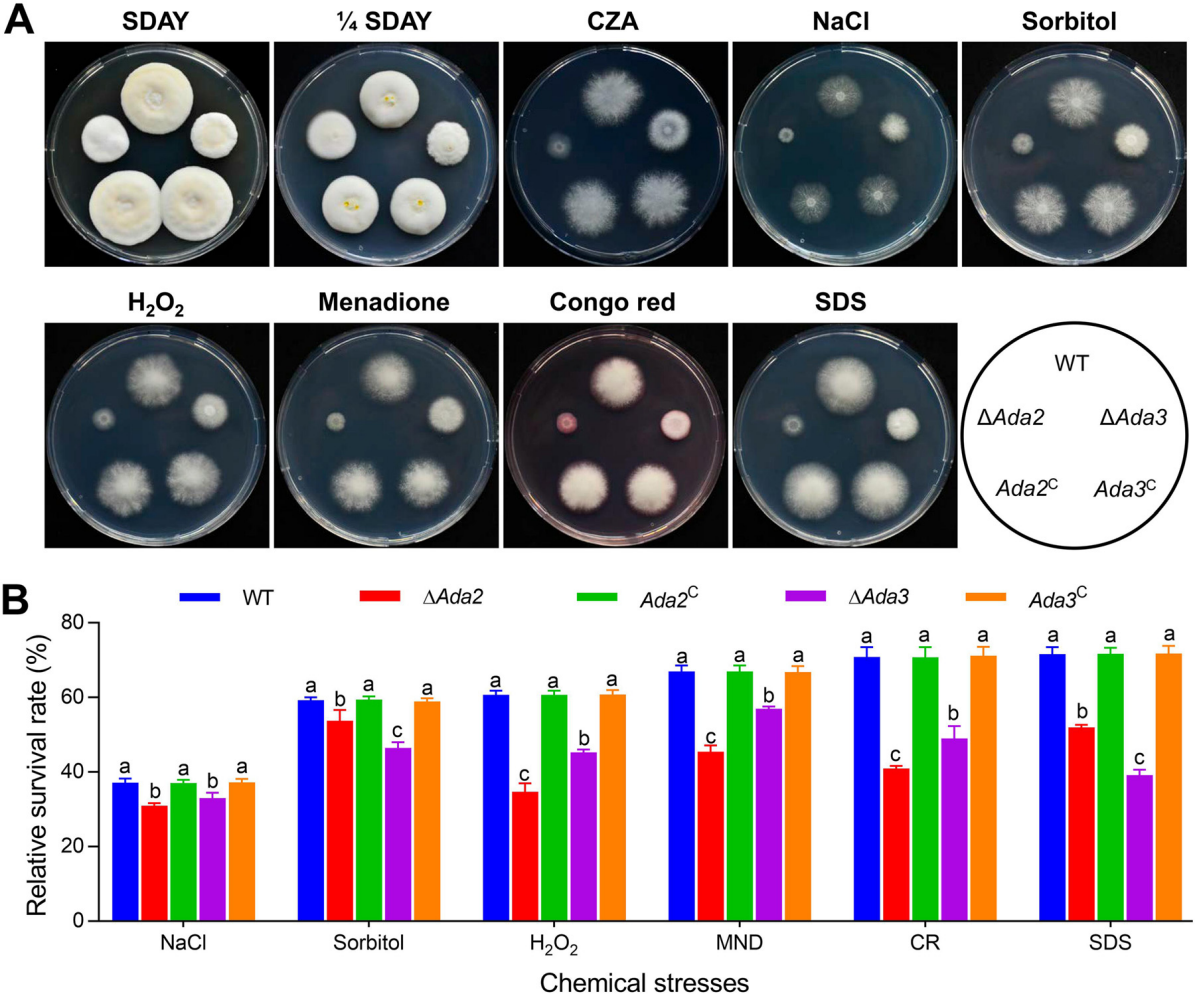
Requirements of *Ada2* and *Ada3* for mycelial growth of B. bassiana under normal and stress conditions. (A) Images of fungal colonies after inoculation of 1 μL suspension (1 × 10^6^ conidia/mL) on SDAY, 1/4 SDAY, CZA, and CZA supplemented with NaCl, sorbitol, H_2_O_2_, MND, CR, or SDS and for 10 days of culturing. (B) Relative survival rates of the strains on CZA plates containing chemical reagents after incubation for 10 days. Different lowercase letters differ significantly among five strains (Tukey’s HSD, *P* < 0.05). Error bars denote SD from three replicates.

The fungal growth of each strain was assessed on SDAY, 1/4 SDAY, CZA (Fig. 1A), and 13 variants CZA media with different nutrients (Table 1). After 10 days of cultivation, the Δ*Ada2* and Δ*Ada3* mutants showed similar defects in mycelial growth on rich SDAY, with the colony area reduced by 59.99% and 58.35% in comparison to wild-type (WT) colonies (*P* < 0.05). On minimal plates containing 1/4 SDAY and CZA, the colony area of the Δ*Ada2* mutant decreased by 37.32% and 83.37%, respectively, and that of the Δ*Ada3* mutant decreased by 44.32% and 46.34%, respectively (*P* < 0.05). On the 13 different CZA-derived media, Δ*Ada2* mutants showed slower growth rates than Δ*Ada3*, decreasing by 78.21% to 94.82% and 31.36% to 83.22%, respectively (*P* < 0.05).

**TABLE 1:** Colony sizes of B. bassiana strains grown on nutrition-rich and limited media for 10 days

Medium^*a*^	Mean (±SD) colony area (cm^2^)^*b*^				
	WT	Δ*Ada2*	*Ada2^C^*	Δ*Ada3*	*Ada3^C^*
SDAY	8.99 ± 0.15^a^	3.63 ± 0.17^b^	9.25 ± 0.10^a^	3.74 ± 0.10^b^	9.34 ± 0.27^a^
1/4SDAY	4.59 ± 0.11^a^	2.83 ± 0.06^b^	4.59 ± 0.29^a^	2.55 ± 0.02^b^	4.65 ± 0.11^a^
CZA	5.38 ± 0.12^a^	0.88 ± 0.03^c^	5.38 ± 0.32^a^	2.88 ± 0.09^b^	5.58 ± 0.24^a^
C deleted	3.92 ± 0.10^a^	0.59 ± 0.04^c^	4.02 ± 0.11^a^	1.34 ± 0.02^b^	3.95 ± 0.14^a^
Chitin as C	5.17 ± 0.12^a^	0.83 ± 0.03^c^	5.27 ± 0.15^a^	1.91 ± 0.04^b^	5.11 ± 0.20^a^
Trehalose as C	4.59 ± 0.11^a^	0.78 ± 0.06^c^	4.65 ± 0.16^a^	3.19 ± 0.09^b^	4.68 ± 0.20^a^
Glycerin as C	3.48 ± 0.04^a^	0.38 ± 0.01^c^	3.57 ± 0.14^a^	2.18 ± 0.06^b^	3.57 ± 0.19^a^
Glucose as C	6.38 ± 0.22^a^	0.49 ± 0.01^c^	6.53 ± 0.13^a^	3.25 ± 0.09^b^	6.26 ± 0.11^a^
Ethanol as C	1.78 ± 0.03^a^	0.28 ± 0.01^b^	1.79 ± 0.09^a^	0.30 ± 0.03^b^	1.85 ± 0.14^a^
Fructose as C	3.92 ± 0.10^a^	0.20 ± 0.02^c^	3.83 ± 0.13^a^	2.64 ± 0.08^b^	3.86 ± 0.10^a^
NaAc as C	2.75 ± 0.09^a^	0.49 ± 0.01^c^	2.80 ± 0.11^a^	1.19 ± 0.06^b^	2.69 ± 0.15^a^
CZP-N	5.24 ± 0.12^a^	0.58 ± 0.02^c^	5.29 ± 0.16^a^	1.77 ± 0.01^b^	5.38 ± 0.24^a^
NaNO_2_ as N	5.36 ± 0.12^a^	0.49 ± 0.01^c^	5.49 ± 0.17^a^	2.69 ± 0.01^b^	5.35 ± 0.15^a^
NH_4_NO_3_ as N	4.97 ± 0.11^a^	1.07 ± 0.03^c^	5.07 ± 0.21^a^	2.64 ± 0.08^b^	5.13 ± 0.24^a^
NH_4_Cl as N	4.84 ± 0.11^a^	0.80 ± 0.02^c^	5.11 ± 0.20^a^	2.78 ± 0.09^b^	5.19 ± 0.24^a^
C&N deleted	3.57 ± 0.10^a^	0.60 ± 0.03^b^	3.65 ± 0.20^a^	0.66 ± 0.04^b^	3.69 ± 0.26^a^

a All amended CZA media were prepared with indicated sources.

b Means followed by different lowercase letters in each line are significantly different (Tukey’s HSD, *P* < 0.05).

The Δ*Ada2* and Δ*Ada3* mutants exhibited increased sensitivity to osmotic, oxidative, and cell wall perturbing stresses during colony growth on CZA medium containing NaCl, sorbitol, H_2_O_2_, menadione (MND), Congo red (CR), and sodium dodecyl sulfate (SDS), respectively (Fig. 1A and B). As indicated by the relative survival rate, the tolerance of Δ*Ada2* to the above chemical stresses was reduced by 16.67%, 9.22%, 42.83%, 32.13%, 42.20%, and 27.39%, respectively, while the resistance of Δ*Ada3* was decreased by 11.10%, 21.55%, 25.34%, 14.91%, 30.82%, and 45.21%, respectively (*P* < 0.05). These results suggest important roles of *Ada2* and *Ada3* in maintaining stress resistance and environmental fitness in B. bassiana.

### Ada2 and Ada3 contribute to asexual development.

**FIG 2:**
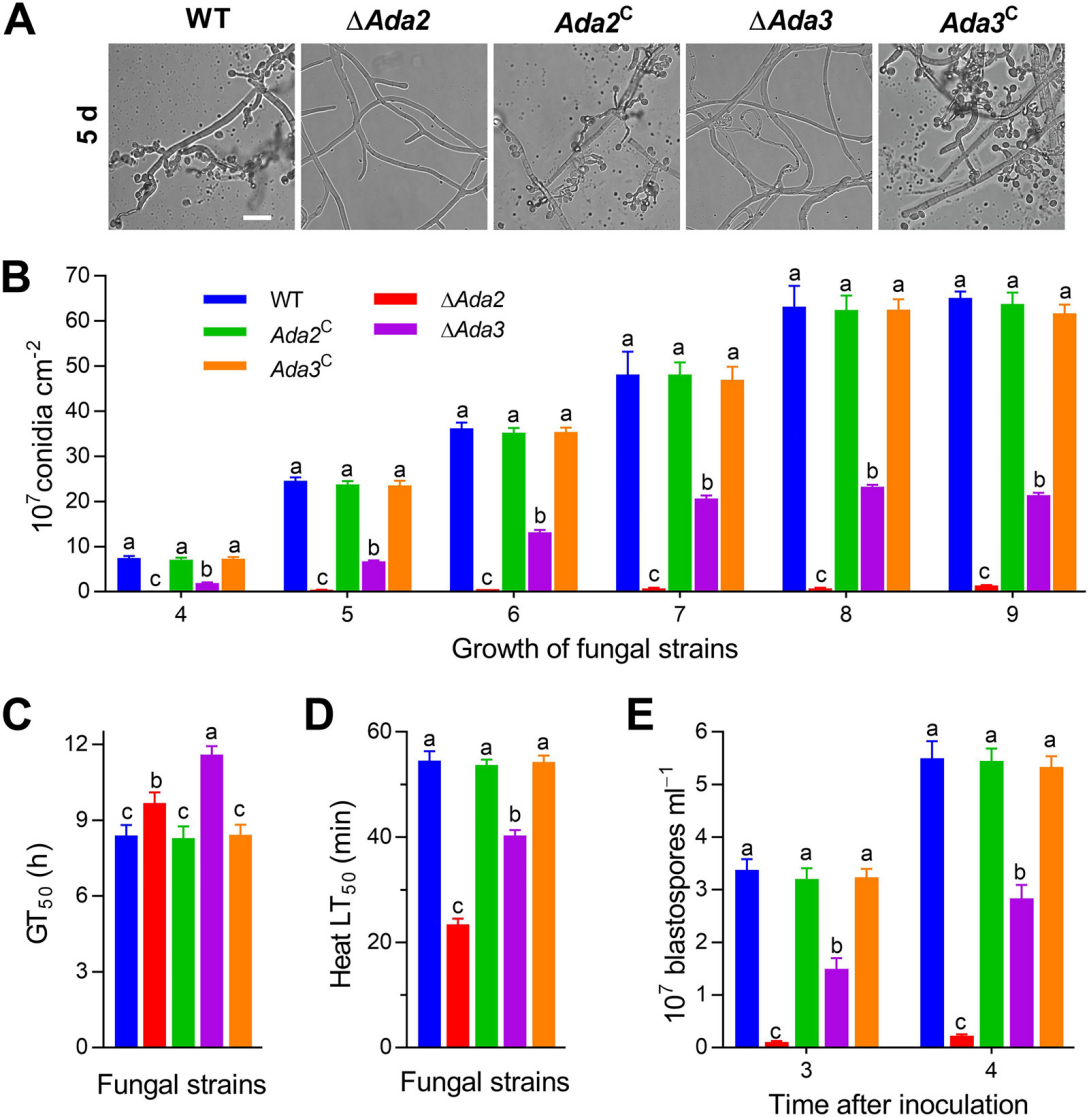
Influences of *Ada2* and *Ada3* on the yield and quality of asexual spores of B. bassiana. (A) Images of conidiation and spore balls in Δ*Ada* and control strains incubated on SDAY plates for 5 days (scale bars, 10 μm). (B) Determination of the conidial production of different strains on CO-SDAY plates over 4 to 9 days under 12:12 L:D cycles at 25°C. (C) The time of median germination (GT_50_, h) evaluated to assess the viability of fungal conidia at 25°C. (D) The median lethal time (LT_50_, min) estimated to assess the tolerance of fungal conidia at 45°C. (E) Quantification of blastospore production in fungal strains after 3 and 4 days of incubation in NLB liquid culture. Different lowercase letters differ significantly among five strains (Tukey’s HSD, *P* < 0.05). Error bars denote SD from three replicates.

A 100-μL conidial suspension was inoculated onto CO-SDAY at 25°C for 9 days of culturing to observe conidial production in different strains. At 5 days of culture, the controls (WT and complemented strains) formed some spore balls and a large number of conidia. By comparison, conidia were rarely observed in Δ*Ada2* and Δ*Ada3* cultures (Fig. 2A). Within 4 to 9 days of culture, conidiation capacity was almost completely suppressed in the Δ*Ada2* mutant. In the Δ*Ada3* mutant, the conidial number was significantly reduced by 57.07% to 73.99% (Fig. 2B; *P* < 0.05). A decrease in blastospore yields was observed in NLB cultures of the Δ*Ada2* and Δ*Ada3* mutants (Fig. 2E; *P* < 0.05). Compared with WT, deletion of *Ada2* or *Ada3* resulted in a significant decline in blastospore yields of 96.79% and 54.59% at 3 days and 95.85% and 46.58% at 4 days, respectively.

In addition to reduced conidial yield, conidial viability and quality were severely impaired in the Δ*Ada2* and Δ*Ada3* mutants. In a germination experiment, the time for 50% conidial germination (GT_50_) was increased to 9.68 ± 0.42 h and 11.6 ± 0.33 h for Δ*Ada2* and Δ*Ada3*, which was 1.15 times and 1.38 times higher than that for WT in GB (Fig. 2C; *P* < 0.05). The thermal resistance of the conidia of different strains to wet heat at 45°C is represented by the median lethal time (LT_50_), and the LT_50_ of Δ*Ada2* and Δ*Ada3* was decreased by 57.06% and 26.13%, respectively, compared to that of the WT (Fig. 2D; *P* < 0.05).

### Ada2 and Ada3 contribute to fungal virulence.

**FIG 3:**
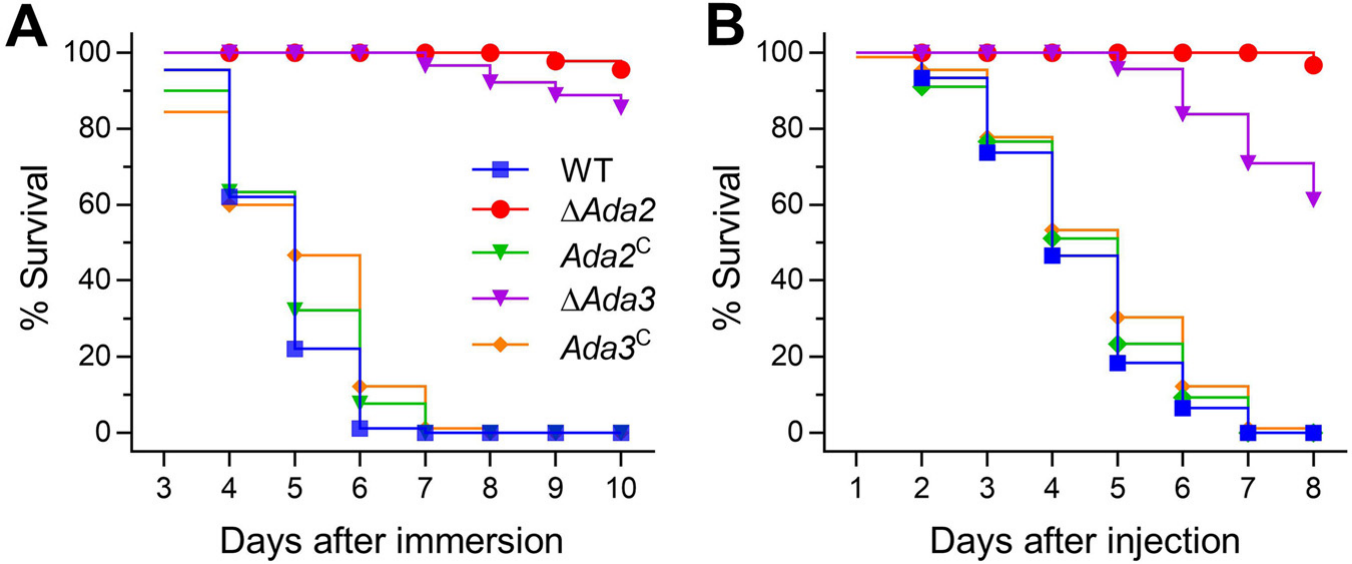
Indispensability of *Ada2* and *Ada3* for the virulence of B. bassiana. (A and B) Kaplan-Meier survival curves estimating the survival trend of G. mellonella treated by immersion in conidial suspension (1 × 10^7^ conidia/mL) or injection of approximately 500 conidia into each larval hemocoel. The death of the larvae treated with different strains was recorded every 24 h during the observation period.

**FIG 4:**
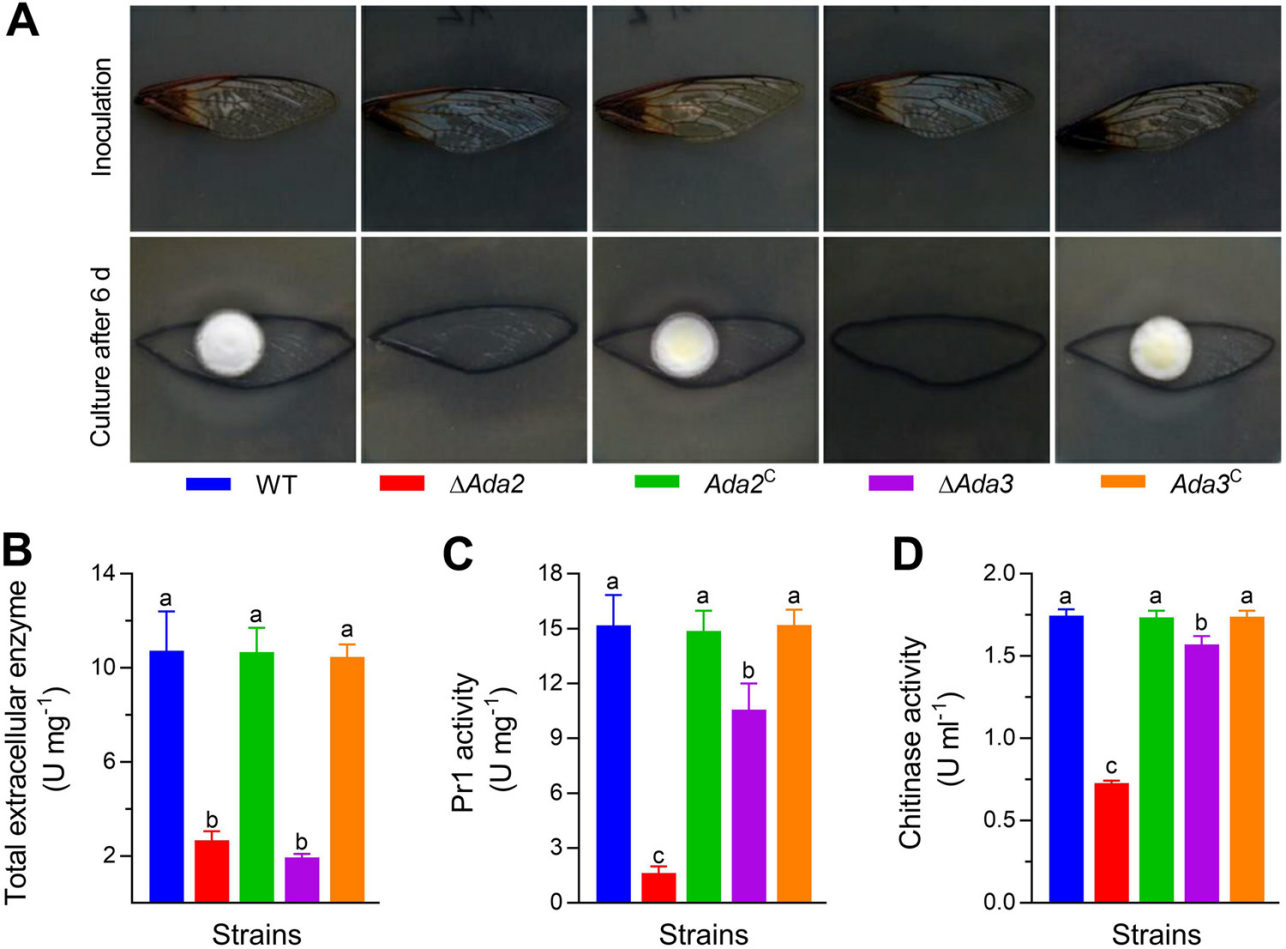
Contributions of Ada2 and Ada3 to virulence-related cellular events in B. bassiana. (A) Images of fungal conidia inoculated on cicada wing sticks on SDAY medium for 3 days; and the fungal conidia remaining on the medium were cultivated for another 3 days after removal of the wings. (B and C) The extracellular enzyme and Pr1 protease activities of different strains measured after 2 days of culture in CZB-BSA induction medium supplemented with 0.5% bovine serum albumin. (D) Chitinase activity was measured by a colorimetric method after 2 days of cultivation in CZB (omitted C and N sources) containing cicada cuticles. Different lowercase letters differ significantly among five strains (Tukey’s HSD, *P* < 0.05). Error bars denote SD from three replicates.

Fourth-instar Galleria mellonella larvae were used in the bioassays to determine virulence, and Kaplan-Meier curves were established to analyze the survival data (Fig. 3A and B). After topical inoculation, the larvae treated with WT died completely by day 7, while the mutant strain did not kill the larvae. After 10 days of infection, larvae in the Δ*Ada2* treatment group showed a survival rate of 95.66%, and the survival rate of larvae in the Δ*Ada3* treatment group was 85.56%. In the cuticle penetration bioassay, larvae treated with the Δ*Ada2* or Δ*Ada3* mutant were still alive on day 4, whereas larvae treated with WT had a mortality of 53.47%. By day 8, the mortality of larvae treated with Δ*Ada2* or Δ*Ada3* reached 3.27% and 38.70%, respectively, and all the larvae treated with the control were dead. In a fungal penetration assay, the Δ*Ada2* and Δ*Ada3* mutants were unable to develop colonies after 3 days of cultivation without cicada wings, while the WT could form colonies (Fig. 4A). To explore possible reasons for the almost complete loss of virulence of Δ*Ada2* and Δ*Ada3* during cuticular infection, some critical enzymes, including extracellular enzymes, Pr1 protease, and chitinase, were examined. After 2 days of culture in CZB-BSA, the extracellular enzyme activity and Pr1 protease activity produced by the same amount of hyphae were reduced in Δ*Ada2* by 75.18% and 89.29%, respectively, and those in Δ*Ada3* were reduced by 82.01% and 37.02%, respectively, relative to those in WT (Fig. 4B and C; *P* < 0.05). Similarly, chitinase activity in Δ*Ada2* and Δ*Ada3* mutants was also significantly reduced by 58.22% and 10.02%, respectively (Fig. 4D; *P* < 0.05).

### Both Ada2 and Ada3 are required for the HAT activity of Gcn5.

**FIG 5:**
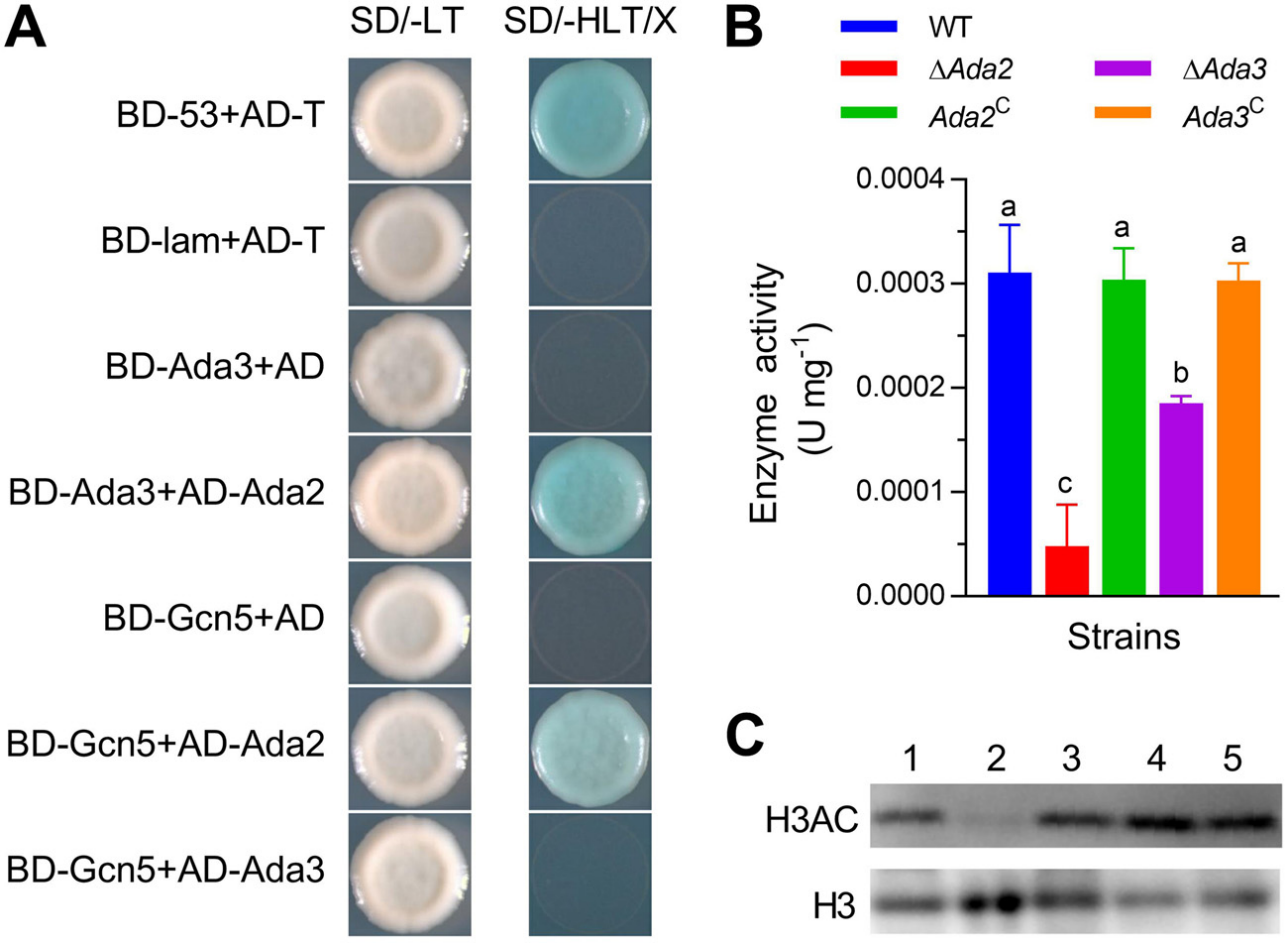
Interactions among Ada2, Ada3, and Gcn5. (A) Yeast two-hybrid tests were applied to analyze the interaction of Ada2 with Ada3 (AD-Ada2 BD-Ada3), Ada2 with Gcn5 (AD-Ada2 BD-Gcn5), or Ada3 with Gcn5 (AD-Ada3 BD-Gcn5). The Y2HGold yeast strain contained AD-T and BD-53, and AD-T and BD-lam were the positive and negative controls, respectively. The experimental results were observed on synthetic dextrose minimal medium supplemented with X-*a*-Gal without histidine, leucine, and tryptophan (SD/-HLT/X) plates. (B) Gcn5 HAT activity was determined by spectrophotometry, and each strain was cultured on SDAY medium for 4 days. (C) Western blot analysis of the acetylation level of histone H3 in the Δ*Ada* mutants and control strains incubated on SDAY for 4 days. Different lowercase letters differ significantly among five strains (Tukey’s HSD, *P* < 0.05). Error bars denote SD from three replicates.

A yeast two-hybrid test was applied to confirm the relationship among Ada2, Ada3, and Gcn5. The Y2HGold yeast strain containing the plasmids AD-Ada2 and BD-Ada3 or AD-Ada2 and BD-Gcn5 grew well on SD-His-Leu-Trp medium supplemented with X-α-Gal. These results demonstrated direct interactions between Ada2 and Gcn5 and between Ada2 and Ada3 (Fig. 5A), indicating the existence of Ada3-Ada2-Gcn5 trimers in B. bassiana. Gcn5 catalytic activity was determined by Western blotting and a fungal/yeast Gcn5-Hat assay kit. Protein extracts from cultures grown for 4 days on SDAY were used to measure Gcn5 catalytic activity. The fungal/yeast Gcn5-Hat assay kit results showed that deletion of either *Ada2* or *Ada3* resulted in 84.56% and 40.35% decreases in Gcn5 activity, respectively (Fig. 5B; *P* < 0.05). This result was similar to those of a Western blot analysis showing decreased histone H3 acetylation levels in the Δ*Ada2* mutant (Fig. 5C). However, reduced histone H3 acetylation was not observed in the Δ*Ada3* mutant.

## DISCUSSION

**FIG 6:**
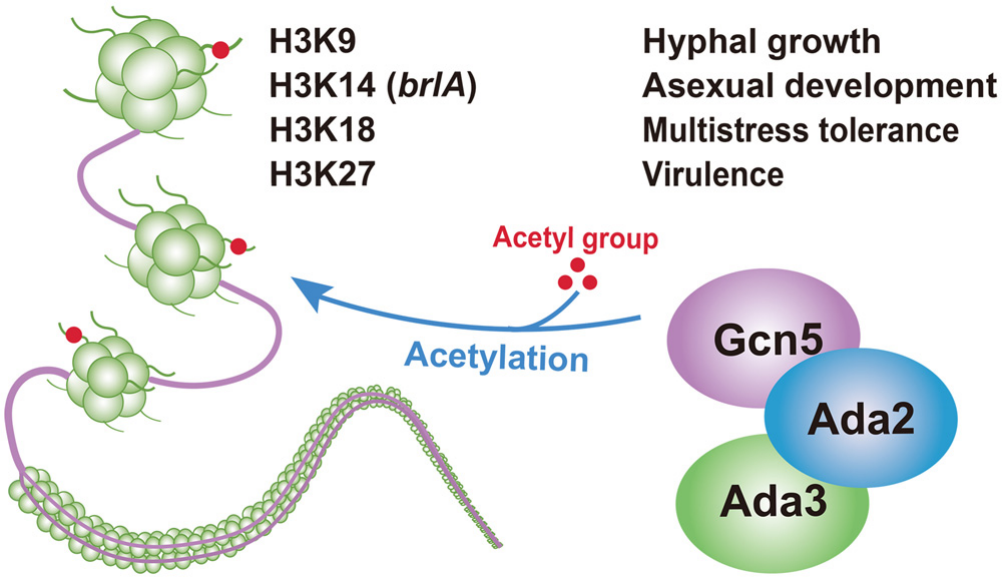
Mechanism of action of the Ada2-Ada3-Gcn5 trimer complex in B. bassiana.

Protein acetylation/deacetylation is considered to be the confluence point for the control of fungal infection (35). A representative HAT, Gcn5, is required for histone acetylation and has been found to modulate key virulence determinants of successful infection by pathogenic fungi in humans, plants, and insects (34, 36–38). Here, we found that Ada2 and Ada3 are functionally linked to Gcn5, forming Ada3-Ada2-Gcn5 trimeric complexes present in B. bassiana (Fig. 6). Moreover, deletion of *Ada2* or *Ada3* reduced Gcn5 enzymatic activity and affected fungal hyphal growth, asexual development, multiple stress tolerances and fungal pathogenicity.

In yeast, SAGA and ADA complexes contain Gcn5, Ada2, and Ada3, which form a ternary complex (24, 39, 40). In the present study, our yeast two-hybrid experiment may indicate that the same Ada3-Ada2-Gcn5 trimer complex exists in B. bassiana. However, *F. fujikuroi* contains a slightly different Gcn5/Ada2/Ada3 trimer complex, with a weak interaction between Ada2 and Ada3 (33). Moreover, simultaneous deletions of two of these three genes do not result in phenotypic defects that are more severe than those of a single deletion in yeast (41, 42). Sgf29, a fourth component of the HAT module, is not required for SAGA complex acetylation of histones or nucleosomes *in vitro* (43). Ada2/Ada3/Gcn5 is the minimal complex for nucleosome acetylation and has been shown to possess the same lysine specificity as the intact SAGA complex (21). In the minimal complex, Ada2 activates the HAT activity of Gcn5 on histone peptides, while Ada3 is responsible for nucleosomal HAT activity (21). In the present study, loss of *Ada2* or *Ada3* also caused a decrease in Gcn5 enzymatic activity. Meanwhile, Western blotting revealed decreased acetylated histone H3 levels in the Δ*Ada2* and Δ*gcn5* mutants (34). Thus, the yeast-like Ada3-Ada2-Gcn5 trimers work together to influence the acetylation of histone H3 in B. bassiana.

Deletion of *Ada2* or *Ada3* exerts different but important influences on the asexual cycle of B. bassiana. First, both *Ada* genes are involved in hyphal growth, and the growth defects of Δ*Ada2* and Δ*Ada3* were similar but weaker than that of Δ*gcn5* on rich SDAY medium (34). This result indicates that *gcn5* makes a more significant contribution to fungal hyphal growth under nutrient-rich conditions. In minimal CZA and a variety of derived media, the Δ*Ada2* mutant had growth defects similar to those of the Δ*gcn5* strain, whereas Δ*Ada3* had less severe growth defects. Therefore, *Ada2* and *Ada3* may play different roles in fungal growth under nutrient-limiting conditions. Previously, the growth defects of *ada2* (*adaB*) or *ada3* deletion strains were found to be strong in A. nidulans and *F. fujikuroi* but not significant in C. albicans and C. neoformans (27, 30, 31, 33). The relative importance of the two *Ada* genes in growth may be different in different fungal species. In the present study, a slower germination rate and a difference in nutrient utilization were the causes of the growth defects of the Δ*Ada2* and Δ*Ada3* mutants. Then, deletion of *Ada2* and *Ada3* nearly abolished and significantly reduced conidial production capacity, respectively. In *F. fujikuroi*, Δ*ada2* and Δ*ada3* mutants lose the ability to form conidia (33). Similar to the Δ*Ada2* strain, the conidiation capacity of B. bassiana was greatly inhibited after deletion of *gcn5* (34). In contrast, deletion of *Mrada3* or *MrGcn5* led to a significant increase in the conidial yield in *M. ruber* M7 (32, 44). Usually, the conidiation process is regulated by the successive expression of *BrlA*, *AbaA*, and *WetA* within the central developmental pathway (CDP) (45). The absence of the master regulator *brlA* results in the inability of B. bassiana to produce conidia (46). The expression of *brlA* is activated not only by the fluffy genes *flbA* to *flbE* and *fluG* and the zinc finger protein BbSmr1 but also by the Gcn5-catalyzed acetylation of histone H3K14 at vital sites in the *brlA* promoter region in B. bassiana (34, 47, 48). In B. bassiana, deletion of *Ada2* or *Ada3* reduced Gcn5 enzyme activity, which inhibited *brlA* transcription and impeded CDP, thereby reducing fungal conidial production (34).

In C. albicans, *Ada2* has an important influence on virulence by affecting the tolerance of the fungus to oxidative stress (27). The absence of *NGG1* significantly affects virulence by decreasing stress adaptation and inhibiting filamentous growth and biofilm formation (29). Our results also demonstrated the indispensability of *Ada2* and *Ada3* in host infection and their role as vital virulence factors in B. Bassiana. First, deletion of *Ada2*, *Ada3*, or *gcn5* severely compromised virulence against G. mellonella during cuticle infection. The insect cuticle is the first obstacle in combating fungal infection and is mainly composed of proteins and chitin (49). Fungi secrete a variety of extracellular enzymes, including Pr1 proteases and chitinases, that decompose the structural components of the cuticle into C and N sources for fungal growth and facilitate fungal penetration (50). In this study, a dramatic decline in the activity of a series of hydrolytic enzymes may also be the reason for the decreased virulence of the cuticle infection in Δ*Ada2* and Δ*Ada3*. The weaker penetrance of Δ*Ada* mutants caused the loss of most prerequisites for fungal infection in insects (51). Previously, many studies have demonstrated a tight link between conidial germination and hyphal extension and fungal cuticle infection (2, 52, 53). Attenuated virulence may be the result of a delayed germination rate and a reduced ability to grow hyphae in the Δ*Ada* mutants on minimal media (Table 1). On the other hand, deletion of *Ada2* or *Ada3* also significantly reduced the lethal action following infection by injection. The contribution of *gcn5* to virulence was intermediate between those of *Ada2* and *Ada3* based on the mortality rate. The impairment of the Δ*Ada* and Δ*gcn5* mutants in G. mellonella cuticle-bypassing infection is likely an outcome of decreased blastospore yield. The dimorphic change between hyphae and yeast-like cells determines the rate of mycosis development and host death (54). This dimorphic transition is also dependent on the CDP gene *brlA* in B. bassiana (46). However, deletion of *SGF29* increases the production of morphological variants with a switch to hypervirulence in C. neoformans (55). Additionally, Δ*Ada* mutants are highly sensitive to multiple stress responses, such as heat shock, cell wall and oxidative stresses, which have been shown to be closely related to biocontrol potential (56).

In conclusion, our findings reveal that Ada2 and Ada3 modulate fungal hyphal growth, asexual development and virulence by affecting Gcn5 enzyme activity and histone H3 acetylation in B. bassiana. These findings provide a new understanding of the life cycle of entomopathogenic fungi and the function of the Gcn5 acetyltransferase complex in pathogens. Future studies of proteins or DNA regions that physically interact with Ada or other components of the Gcn5-containing HAT complex will enable us to further decipher the histone acetylation mechanism of B. bassiana.

## MATERIALS AND METHODS

### Microbial strains and culture conditions.

B. bassiana ARSEF 2860 (WT) was obtained from the RW Holley Center for Agriculture and Health (Ithaca, NY, USA) and stored at −80°C. Normal growth of B. bassiana was cultured in rich SDAY (4% glucose, 1% peptone, 1% yeast extract plus 1.5% agar) plates at 25°C under a 12:12 h light/dark cycle.

### Construction of *Ada2* and *Ada3* mutants.

The genes *Ada2* and *Ada3* in the WT strain were disrupted individually by homologous replacement as previously described (57). The 5′- and 3′-flanks of the targeted genes were cloned with primers (Table S1), dealt with specific restriction enzymes, and then inserted into the corresponding sites of the plasmid. Agrobacterium tumefaciens AGL-1 was transformed with the resultant plasmids, p0380-bar-Ada2 and p0380-bar-Ada3, for gene deletion, and *bar* resistance to phosphinothricin was used to screen putative deletion mutants (200 μg/mL) (58). Full-length *Ada2* and *Ada3* sequences were inserted into the vector p0380-sur-gateway to synthesize the complementation plasmids. The plasmids provided resistance to chlorimuron ethyl for screening the putative complementation strains (10 μg/mL). After verification by PCR, qRT-PCR was utilized to validate the putative mutants. PCR and qRT-PCR were run with paired primers (Table S1).

### Experiments for fungal growth under different nutrient and stress conditions.

For measurement of fungal growth capability, 1 μL suspension (1 × 10^6^ conidia/mL) of the Δ*Ada2* and Δ*Ada3* strains and the controls was spotted onto plates containing different nutrients, including SDAY, 1/4 SDAY (containing one-fourth of the nutrients in SDAY), CZA medium (3% sucrose, 0.3% NaNO_3_, 0.1% K_2_HPO_4_, 0.05% KCl, 0.05% MgSO_4_, 0.001% FeSO_4_, and 1.5% agar), and 13 amended CZA media. The CZA media were obtained by omitting the carbon source and/or nitrogen source, or by replacing the sucrose with chitin, trehalose, glycerin, glucose, ethanol, fructose, or NaAc, or NaNO_3_ with NaNO_2_, NH_4_NO_3_, or NH_4_Cl. After a 10-day incubation, the diameter of different colonies was cross-measured and converted to colony area (cm^2^) as a growth index.

The same spotting method was applied to evaluate the tolerance of different strains to chemical stressors. The conidial suspension was incubated on CZA (control) plate or CZA supplemented with NaCl (0.5 M), sorbitol (0.6 M), H_2_O_2_ (2 mM), MND (0.02 mM), CR (10 μg/mL), and SDS (80 μg/mL) for 10 days. The relative survival rate was used to evaluate the stress resistance, which represents the ratio of colony area under a given stress to the colony area of the controls.

### Determination of fungal asexual development.

A 100 μL of 1 × 10^7^ conidia mL^−1^ suspension (unless otherwise noted, the concentration was 1 × 10^7^ conidia/mL) was spread onto SDAY plates, covered with cellophane (CO-SDAY), and cultured for 9 days to measure conidiation capacity as previously described (57). The conidial yield was counted and is presented as the number of conidia per cm^2^.

For observation of blastospore yield, 100 μL of conidial suspension was inoculated in 50 mL of nitrogen-limited broth (NLB: 4% glucose, 0.4% NH_4_NO_3_, 0.3% KH_2_PO_4_, and 0.3% MgSO_4_) with continuous shaking at 150 rpm (59). After 3 and 4 days of culture, blastospore production (cell mL^−1^) was recorded. A conidial germination assay was performed to assess the time for 50% conidial germination (GT_50_, h) as a viability index (60). Additionally, the conidial thermotolerance of the strains at 45°C was measured, and the median lethal time (LT_50_, min) was used as the resistance index.

### Detection of fungal virulence and related cellular events.

A model insect, G. mellonella, was used for the bioassay, and the virulence of each strain was assessed by two modes of infection as previously described (57). The treated larvae were placed at 25°C, during which their survival rate was recorded every 24 h. The larval survival data were analyzed using Kaplan-Meier curves.

To further investigate the causes of the impaired virulence in *Ada* mutants, fungal penetration and the activity of cuticle-degrading enzymes, including proteinase and chitinases, were measured as previously described (61, 62). First, 3 μL suspension (1 × 10^5^ conidia/mL) was inoculated on the surface of a cicada wing covered on SDAY medium for 3 days to imitate the fungal penetration process. After removal of the cicada wing, the remaining plates were cultivated for another 3 days to observe the growth of the different strains. Then, a suspension of 100 μL was inoculated into 50 mL CZB (agar-free CZA) supplemented with 0.5% bovine serum albumin (BSA) as the sole nitrogen source. After 2 days of culture at 120 rpm, the cultures were centrifuged at 4°C for 1 min, and the mycelium was collected and weighed after drying overnight at 50°C. The supernatant was used to quantify extracellular enzyme activity and Pr1 protease activity at OD_410_ or OD_440_, respectively. The amount of enzyme required to increase the OD_410_ or OD_440_ value by 0.01 within 1 h compared with the controls was considered to be one unit of activity and converted to U/mg (61). Finally, chitinase activity was measured by a previous method with one modification (62). Mycelia were cultured in CZB for 2 d, and the supernatant was collected to determine chitinase activity.

### Yeast two-hybrid analysis.

The interaction relationship among Ada2, Ada3, and Gcn5 was probed by a yeast two-hybrid assay. The *Ada2* and *Ada3* sequences were amplified from cDNAs and ligated into the prey vector pGADT7 (AD) at the NdeI + BamHI sites. The cloned *Ada3* and *Gcn5* sequences were inserted into the same site in the bait vector pGBKT7 (BD). After sequencing, the verified plasmids BD-Ada3 and AD-Ada2, BD-Gcn5, and AD-Ada2 or AD-Ada3 were then co-transformed into the Y2HGold yeast strain. Meanwhile, yeast was co-transformed with BD-53 and AD-T vectors as a positive control, with BD-lam and AD-T vectors as a negative control. The transformants were observed on SD-His-Leu-Trp medium with the addition of X-α-Gal.

### Assay of Gcn5 enzyme activity and Western blotting to detect the histone H3 acetylation level.

Different strains were spread as 100-μL conidial suspensions on CO-SDAY and cultured for 4 days. The extraction of total protein and the determination of enzyme activity were performed according to the instructions of a fungal/yeast Gcn5-Hat assay kit (Genmed, America). A centrifuge tube was filled with mycelia, 250 μL reagent A, and 100 mg reagent B and shaken for 15 s in an ice bath for 1 min. After repeated shock and incubation in an ice bath 10 times, the mycelia were treated with 250 μL reagent A again. The supernatant was obtained by centrifugation at 4°C for 15 min, and the protein concentration was determined with the kit. Then, 100 μL of C reagent, 40 μL of D reagent and E reagent were added and incubated for 3 min at 30°C. Then, 20-μL samples were used to detect absorbance 6 times over a 10-min period. Except for the addition of 80 μL reagent C and 20 μL reagent F, the nonspecific activity of the samples was determined as described above. Specific enzyme activity is total enzyme activity minus nonspecific enzyme activity. Under the conditions of 30°C and pH 7.4, the amount of enzyme required for reducing 1 μmol NAD per 1 min was taken as a unit of activity.

Mycelia were cultured under the same conditions, and the level of acetylated histone H3 was measured by Western blotting. The hyphae were ground into powder, suspended in 1 mL PBS with pH 7.4, followed by ice bath with protease inhibitor (Mei5 Biotech, Beijing, China) for 10 min. The mixture was centrifuged repeatedly at 4°C to obtain the supernatant, and its protein concentration was measured. The histone H3 levels and acetylated histone H3 levels of different strains were probed using diluted anti-histone H3 (Abcam, catalog #ab1791) and anti-acetyl-histone H3 (Merck Millipore, catalog #06-599) antibodies, respectively.

### Statistical analysis of the experimental data.

The phenotypic estimates determined by one-way ANOVA were obtained from three replicates. Tukey's honest significant difference (HSD) test was used to differ the significance between the Δ*Ada* mutants and their control strains.
