# Supplementary material for: Ada2 and Ada3 Regulate Hyphal Growth, Asexual Development, and Pathogenicity in Beauveria bassiana by Maintaining Gcn5 Acetyltransferase Activity
Source: Microbiol Spectr. 2023 Apr 13;11(3):e00281-23. doi: 10.1128/spectrum.00281-23 (PMC10269768; doi:10.1128/spectrum.00281-23)
Supplement: Supplemental file 1 — Supplemental material. Download spectrum.00281-23-s0001.pdf, PDF file, 0.9 MB [file spectrum.00281-23-s0001.pdf]

Supplemental Material

Ada2 and Ada3 regulate hyphal growth, asexual development, and pathogenicity in *Beauveria bassiana* by maintaining Gcn5 acetyltransferase activity

Shun-Juan Hu[1], Hao Zheng[1], Xin-Peng Li[1], Zhi-Xing Li[1], Chao Xu[1], Juan Li[2], Jia-Hua Liu[1], Wen-Xiao Hu[1], Xian-Yan Zhao[1], Juan-Juan Wang[2], Lei Qiu[1*]

[1] *State Key Laboratory of Biobased Material and Green Papermaking, Qilu University of Technology, Shandong Academy of Sciences, Jinan, China*

[2] *School of Biological Science and Technology, University of Jinan, Jinan, China*

[*]Correspondence author

***Lei Qiu**: State Key Laboratory of Biobased Material and Green Papermaking, Qilu University of Technology, Shandong Academy of Sciences, Jinan, Shandong 250353, PR China. E-mail:* *qiulei.2005@163.com*

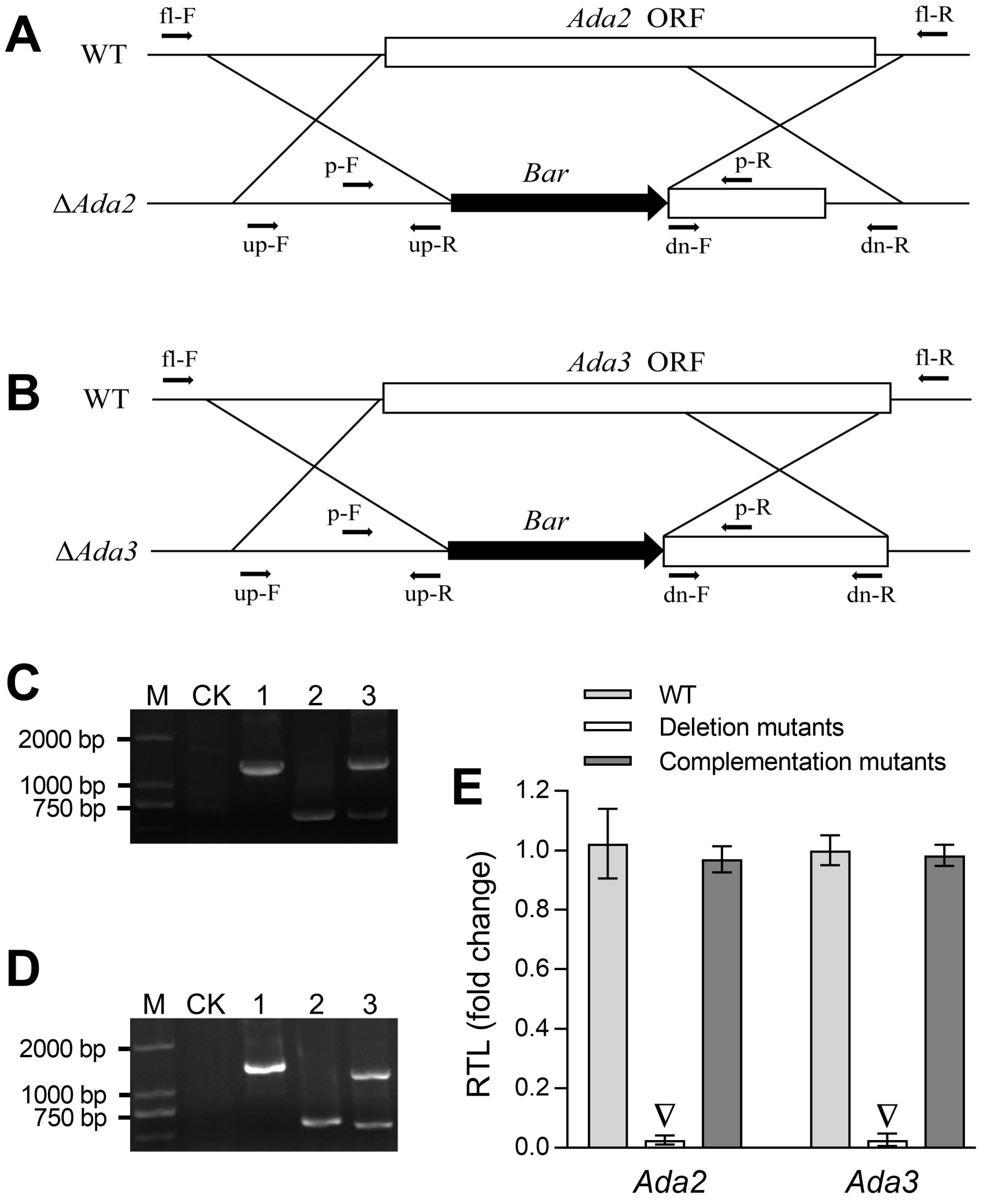

FIG S1 Construction and identification of the Δ*Ada2* and Δ*Ada3* mutants of *B. bassiana*.

(A, B) Schematic diagrams of the strategy for construction of gene disruption and complementation strains. The *Ada2* (C) and *Ada3* (D) mutants were identified via PCR with primer pairs (Table S1). Lane 1: gene disruption mutants. Lane 2: WT. Lane 3: gene complementation mutants. (E) Relative transcript levels (RTL) of *Ada2* and *Ada3* in their deletion mutants and complementation mutants versus the WT standard. Note that RTL of *Ada2* and *Ada3* was undetectable in the deletion mutants (arrowed).

Table S1. Paired primers designed for vector construction, gene deletion and complement.

Primers	Paired sequences (5′-3′)*	Purpose
Ada2up-F/R	AAAAAGAATTCTTGAAGCGGACAAGTGGTTT / AAAGGATCCCTCAATGGTTGCTGTGCTGA	Cloning 5′ *Ada2*
Ada2dn-F/R	AAAAAACTAGTTTGCTTTGCCAAGGGAGAGTC/ AAAAAGTTAACTGAACTAAAGCGCAAGAAACCC	Cloning 3′ *Ada2*
Ada2fl-F/R	GGGGACAAGTTTGTACAAAAAAGCAGGCTGGAAAGAGCACGACGAAAG /<br>GGGGACCACTTTGTACAAGAAAGCTGGGTGGTCCCATAAAGAATAGAACAGA	Cloning full-length *Ada2*
pAda2-F/R	AAAAGAGTTTCCCTGTTCTCCTGC / AAACATAGATCTCGGCACCTTCAA	PCR detecting *Ada2*
qAda2-F/R	CTACACAACGCAACAAGC / TGACGACACCATTAGACTG	qRT-PCR detecting *Ada2*
Ada3up-F/R	AAAGAATTCACAGACAAACCTCCGTAAGA / AAAGGATCCGATGGAAAGAATGCGAAA	Cloning 5′ *Ada3*
Ada3dn-F/R	AAACTCGAGTCAAGAGCCTCACTAACATAAT / AAATCTAGAAGAGTCCAAATCCTCCAAA	Cloning 3′ *Ada3*
Ada3fl-F/R	GGGGACAAGTTTGTACAAAAAAGCAGGCTCTGAGTGGATGACGGAGAAT /<br>GGGGACCACTTTGTACAAGAAAGCTGGGTAATACAAAGAGTGGGAAGACAA	Cloning full-length *Ada3*
pAda3-F/R	AAAGCCTTGGGTCATAACTCG / AAAATTTCTTGCCCTCATTCTC	PCR detecting *Ada3*
qAda3-F/R	TGCTACTAACGGATTGAATGG / ATGTGTCTGAGTTCTTGCTT	qRT-PCR detecting *Ada3*
Ada2-AD-F/R	ACCAGATTACGCTCATATGATGGGTGTGATTCGCAAAAAGACT /<br>AGCTCGAGCTCGATGGATCCTCAAGCCTTTCCAACCCAGC	Cloning *Ada2* cDNA for ligating to pGADT7
Ada3-AD-F/R	ACCAGATTACGCTCATATGATGGCACCCGGATCCAGC /<br>AGCTCGAGCTCGATGGATCCCTACTCCTCCTCGGCTTC	Cloning *Ada3* cDNA for ligating to pGADT7
Ada3-BD-F/R	AGAGGAGGACCTGCATATGATGGCACCCGGATCCAGC /<br>CGCTGCAGGTCGACGGATCCCTACTCCTCCTCGGCTTC	Cloning *Ada3* cDNA for ligating to pGBKT7
Gcn5-BD-F/R	AGAGGAGGACCTGCATATGATGTCAGAGATCAAGGAAGAGATAG /<br>CGCTGCAGGTCGACGGATCCTTACTTTTCCGGCTCCAGATGC	Cloning *Gcn5* cDNA for ligating to pGBKT7
pAD-F/R	TGAAGATACCCCACCAAACCC / GTGAACTTGCGGGGTTTTTCAG	PCR detecting AD plasmid
pBD-F/R	TCATCGGAAGAGAGTAGT / AGAGTCACTTTAAAATTTGTATA	PCR detecting BD plasmid

* Underlined regions denote the sites of restriction enzyme in the *ΔAda2* mutant (*Eco*RI/*Bam*HI and *Spe*I/*Hpa*I) and *ΔAda3* mutant (*Eco*RI/*Bam*HI and *Xho*I/*Xba*I), or gateway fragments exchanged for the targeted *Ada2* and *Ada3* complementation mutants.
